# Epigenetic profiles of elevated cell free circulating H3.1 nucleosomes as potential biomarkers for non-Hodgkin lymphoma

**DOI:** 10.1038/s41598-023-43520-0

**Published:** 2023-09-28

**Authors:** Priscilla Van den Ackerveken, Alison Lobbens, Dorian Pamart, Aristotelis Kotronoulas, Guillaume Rommelaere, Mark Eccleston, Marielle Herzog

**Affiliations:** Belgian Volition SRL, 22 Rue Phocas Lejeune, Parc Scientifique Crealys, 5032 Isnes, Belgium

**Keywords:** Non-hodgkin lymphoma, Biomarkers, Post-translational modifications

## Abstract

During cell death, nucleosomes, the basic structural unit of chromatin, are released into the blood stream and elevated levels have been found in the plasma of patients with solid cancers. In this study, we demonstrate an increase in cell free circulating H3.1-nucleosomes levels in plasma samples from patients with hematological malignancy, non-Hodgkin lymphoma (NHL), relative to healthy donors. As histone post-translational modifications (PTMs) of circulating nucleosomes are described as potential biomarkers of various solid cancers, we investigated the epigenetic profile of nucleosomes from NHL patients following nucleosome enrichment (Nu.Q® capture) combined with mass spectrometry. Eight histones PTMs, including the acetylation of histone H3 at lysine 9, 14 and 18 as well as the methylation state of histone H3 at lysine 9, 27 and 36, were identified at a higher level in the plasma of NHL patients compared to healthy donors. These results were confirmed in a larger clinical cohort by immunoassay. Subsequently, the temporal profile of these histone PTMs in NHL patients undergoing treatment course highlighted the potential use of these new biomarkers to monitor treatment response and/or disease progression. Our results substantiate that levels of H3.1-nucleosomes are particularly elevated in NHL patients and may be a useful diagnostic tool. Moreover, our work emphasizes the crucial roles of the epigenetic marks present on circulating nucleosomes to detect and monitor tumor progression and/or treatment response of non-Hodgkin Lymphoma.

## Introduction

Non-Hodgkin lymphoma (NHL) is a common group of hematological malignancies originating from lymphocytes and found predominantly in the lymphoid tissue. The most common symptom of NHL is a painless swelling in the lymph nodes together with other non-specific symptoms such as fever, night sweats, weight loss and tiredness. Diagnosis of NHL is based on a lymph node biopsy. However, due to their invasive nature, tissue biopsies have many limitations especially for monitoring therapeutic response and tumor progression where multiple biopsies may be required. Liquid biopsies offer a simpler, non-invasive alternative to surgical biopsies but may require significant blood volumes to identify tumor derived cell free circulating DNA.

In the nuclei of eukaryotic cells, 147 bp stretches of DNA are wrapped around a core particle of eight histone proteins within nucleosomes and these structural units, connected by linker DNA, form chromatin. The nucleosome structure is dynamically regulated by many epigenetic modulators including the replacement of the histone variants and the post-translational modifications of histones. Among the variants of the histone H3, there are two canonical variants, the H3.1 and H3.2 that is incorporated during DNA replication and non-canonical such as H3.3 that becomes enriched in postimitotic cells. Post-translational modifications, mainly of the N-terminal tails of the H3 histone proteins, dynamically modulate chromatin accessibility in combination with more permanent DNA modifications (e.g. methylation or hydroxymethylation) and provide regulation of critical cell processes such as transcription, DNA replication, DNA repair and ultimately, cell death^1–4^. Disruption of this histone code can lead to altered gene function and malignant cellular transformation. It is well established that the global changes in the epigenetic landscape are a hallmark of cancer. For instance, aberrant DNA methylation has been defined as a central feature of hematologic malignancies^5,6^. Disfunction of epigenetic enzymes, such as DNA methyltransferases and histone modifiers (i.e. Histone acetyltransferases (HATs), histone deacetylases (HDACs) and histone methyltransferases) are frequently identified in NHL^7^. Moreover, acetylation imbalance has been demonstrated to influence gene expression either at the tumor suppressors levels or proto-oncogenes^8–13^.

An increase of circulating cell-free DNA (cfDNA) has been detected in the blood of many cancer subtypes^14–16^ including in NHL patients^17–19^. Additionally, elevated nucleosome levels have been detected in human NHL patients^20^ and in dogs suffering of lymphoma^21^, which can be used as a comparative model of human non-Hodgkin lymphoma^22^. However, the epigenetic marks present on circulating nucleosome have not yet been extensively characterized in this pathology. Recently, several studies have identified differential histone PTMs on circulating nucleosomes in cancer patients^20–22^ suggesting their potential use as cancer biomarkers. These epigenetic analyses of circulating nucleosomes were carried out using singleplex antibody-based approaches, limiting the biomarker discovery process to established assays. To overcome this limitation, we recently developed a novel approach called Nu.Q® Capture—Mass spectrometry (MS) that combines the immunoprecipitation of H3.1 circulating nucleosomes from plasma followed by tandem mass spectrometry (LC–MS/MS) to discover new epigenetic biomarkers^23^. Mass spectrometry-based proteomics is a promising technology for quantitative assessment of proteins and their associated PTMs with a high accuracy and sensitivity^24–27^.

In this study, we identified high levels of H3.1-nucleosomes in plasma of NHL patients compared to healthy donors and these H3.1-nucleosome levels correlated with the level of cfDNA. After which, using the Nu.Q® Capture—MS workflow, we profiled 33 histone PTMs from circulating nucleosomes of NHL samples and identified 8 epigenetic markers able to distinguish between healthy donors and pathologic disease. The subsequent validation of the LC–MS/MS results using specific anti-histone PTMs immunoassays confirmed that the previously identified histone PTMs panel could be used as potential biomarkers for NHL diagnosis as well as monitoring tumor progression and treatment response.

## Methods

### Ethics approval and consent to participate

Informed consent was obtained from all subjects involved in the study and all experiments and methods were performed in accordance with relevant guidelines and regulations. The study followed the principles outlined in the Declaration of Helsinki for all human experimental investigations. All blood specimens were purchased from biobanks where the blood samples are ethically collected with IRB-approved protocols. For NHL patients, the protocol/study number is *PG-ONC 2003/1 for samples coming from DxBioSamples LLC (DxBioSamples LLC, San Diego, California, US) and DLSCA2010112 as well as ABRT-001-v2/DLSSD13 for samples collected by Discovery Life Sciences (Discovery Life Sciences, Huntsville, Texas, USA; protocol/study).* For healthy donors (iNoSpecimens BioBank, Clermont Ferrand, France), the biobank has the authorization to market residual samples from medical analysis to carry out scientific research (file number: AC-2018-3151 and authorization number: IE-2018-978).

### Sample collection and cohort information

*MS biomarker discovery and DNA analysis* K2-EDTA plasma samples from Caucasian healthy donors (n = 5) and NHL patients (n = 9) were obtained from a commercial biobank (DxBioSamples LLC, San Diego, California, US) (Supplementary Table 1). Plasma samples were isolated by centrifugation of whole blood (10 min at 1.500 g at + 4 °C/39 °F) and frozen within 4 h of collection according to the suppliers standard operating procedure.

*Evaluation of the MS Biomarker signature by immunoassay* an independent cohort of NHL patients (n = 24 from DxBioSamples LLC San Diego, California, US) and healthy donors (n = 35 from iNoSpecimens BioBank, Clermont Ferrand, France) was used (Supplementary Table 2).

*The treatment follow-up study* a cohort comprised 2 patients with Diffuse large B cell lymphoma (DLBCL) under chemotherapy (Discovery Life Sciences, Huntsville, Texas, USA). Patient #1, a 47-year-old female received R-CHOP® (Day 1, 32, 42, 63 and 84) combined with Elitek® (Day 1, 42) for the management of plasma uric acid level. Finally, a radiation therapy (Rx) was applied (Day 210 until day 224). For this patient, 4 K2-EDTA plasma samples were collected (Day 14, 63, 84 and 210). Patient #2, a 69-year-old male, received intensive infusion treatment regimen (T1) comprising R-CHOP® and EPOCH-R® with Elitek® (Day 1, 153). A second treatment cycle (T2) comprising Rituximab with Methotrexate and Temodar® (Day 216, 223) was administered followed by a third treatment (T3) with Ruxience administration (Day 307). The patient also received a trial treatment (T4) with Temodar (Day 314). 5 K2-EDTA plasma samples were obtained (Day 153, 223, 307, 321 and 469) (Supplementary Table 4).

*cfDNA extraction* cfDNA from plasma samples were extracted using QIAamp® DSP Circulating NA kit (Qiagen; Antwerpen—Belgium) following manufacturer’s instructions. Briefly, plasma samples (≥ 500µL) were applied to a QIAamp Mini column followed by washing on a vacuum manifold. cfDNA was eluted in ≥ 30 µL of elution buffer for subsequent quantification.

Quantification of cfDNA was performed by Qubit dsDNA High Sensitivity assay (Life Technologies Europe B.V; Merelbeke—Belgium) with a Qubit® 4.0 Fluorometer (Life Technologies Europe B.V; Merelbeke—Belgium) according to the manufacturer’s instructions. The range of quantification was 0.1–120 ng. The amount of cfDNA was then normalized by multiplying the concentration obtained using the Qubit dsDNA kit (ng/µL) by the elution buffer volume used (µL) and divided by the volume of the sample extracted (mL) to obtain a concentration expressed as ng of cfDNA per mL of plasma.

cfDNA size distribution of the plasma sample was assessed using an Agilent 2100 Bioanalyzer (Agilent Technologies; Diegem—Belgium) with an Agilent High Sensitivity DNA Kit for fragment sizes of 50–7000 bp (Agilent Technologies; Diegem—Belgium), according to manufacturer’s instructions. Briefly, samples were loaded on to a microfluidic chip and nucleic acid fragments separated by electrophoresis based on their size.

*Circulating nucleosome immunoprecipitation (Nu.Q® Capture) *M280 Tosylactivated magnetic beads (Life Technologies Europe BV; Oslo—Norway) were coated with an anti-histone H3.1 monoclonal antibody (Belgian Volition SPRL, Isnes, Belgium) as previously described^23^. 1 mg of coated beads were incubated with 900µL of K2-EDTA plasma for 1 h at room temperature on a roller. After incubation, the beads were pelleted on a magnetic separation rack and the captured circulating nucleosomes retained. The supernatant was assessed for nucleosome depletion by immunoassay according to the manufacturer’s instructions (Nu.Q® H3.1, Belgian Volition SRL, Isnes, Belgium). Percentage depletion was defined as 1 − *([nucleosome]*_*supernatant*_*/[nucleosome]*_*total*_*)*. The beads were washed 3 times with wash buffer on a roller and rinsed 4 times with PBS1x to remove nonspecifically bound material and detergent before being dried for 1 h at RT.

### Characterization of Histone post-translational modification by LC–MS/MS

Immunoprecipitated nucleosomes were then processed by EpiQMAx GmbH (Planneg, Germany) to analyze the histone post-translational modification pattern by LC–MS/MS. Immunoprecipitated nucleosomes were propionylated and trypsinized on bead as previously described^23^. Heavy amino acid-labeled peptides (Supplementary Table 5) were added to digested samples for normalization and quantifications purposes. The resulting mixture was separated by liquid chromatography (Ultimate 3000 RSLC nano System (Thermo-Fisher Scientific, San Jose—CA) containing a 15‐cm analytical column (75 μm ID with ReproSil‐Pur C18‐AQ 2.4 μm from Dr. Maisch) directly coupled to an electrospray ionization source and Q Exactive HF mass spectrometer (Thermo-Fisher Scientific, San Jose—CA). A data-dependent acquisition mode was used to automatically switch between full scan MS and MS/MS acquisition. The same chromatographic and MS parameters were used as described previously^23^. For the analysis, the raw peptides of canonical histone peptides and their respective PTMs intensities were searched with Skyline software^28^ and normalized using the intensities of corresponding spiked-in heavy peptides. For peptides without spiked-in heavy standards (e.g., from H4), normalization was done using the overall intensity trend of the heavy standards. For peptides below the limit of detection, an arbitrary value of 1 was assigned as intensity to allow downstream statistical analysis. The ratios of light/ heavy peptide were then normalized over their average value across all samples and the log2 values were calculated.

### Quantification of Histone PTMs and histone variants by immunoassay (Nu.Q® Immunoassay)

Six Histone PTMs (H3K9Me1-, H3K27Me3-, H3K36Me3-, H3K18Ac-, H3K14Ac-, and H3K9Ac-nucleosome) and the total H3.1-nucleosome levels were assessed by immunoassay (Nu.Q® Immunoassays, Belgian Volition SRL, Isnes, Belgium) according to the manufacturer’s instructions. Each sandwich immunoassay was performed on an IDS-i10 automated chemiluminescence immunoanalyzer system (Immunodiagnostic Systems Ltd (IDS) UK) using magnetic beads technology. Briefly, 50 μL of K2-EDTA plasma was mixed with an acridinium ester labeled anti-nucleosome detection antibody. Then, an anti-histone modification/variant antibody coated beads were added. Finally, trigger solutions were added following a wash step and the signal emitted was measured by the luminometer. Results were expressed in RLU (Relative light Unit), and the concentrations were evaluated using a four-parameter logistic regression of a reference standard curve. If the %CV of the determined concentration was above 20%, the analysis was repeated. The fold change of histone PTMs represents a ratio which is computed as the level of specific circulating nucleosomes measured on NHL samples divided by the median level of the reference healthy samples defined in Sect. 3.3.

### Statistical analysis

Heat map and Box and whisker plots showing the min to max distribution and the PCA (Principal Component Analysis) analysis were performed using GraphPad InStat software (GraphPad Software, USA) using Log2 normalized ratios (defined as ratios of relative abundances normalized over the average value of the given PTM across all samples). The Mann–Whitney test was used for unpaired comparisons and one-way ANOVA Test was used for multiple comparisons (*p < 0.05; **p < 0.01; ***p < 0.001).

## Results

### Elevated levels of circulating H3.1-nucleosomes are associated with NHL and correlated to cfDNA levels

**Figure 1:**
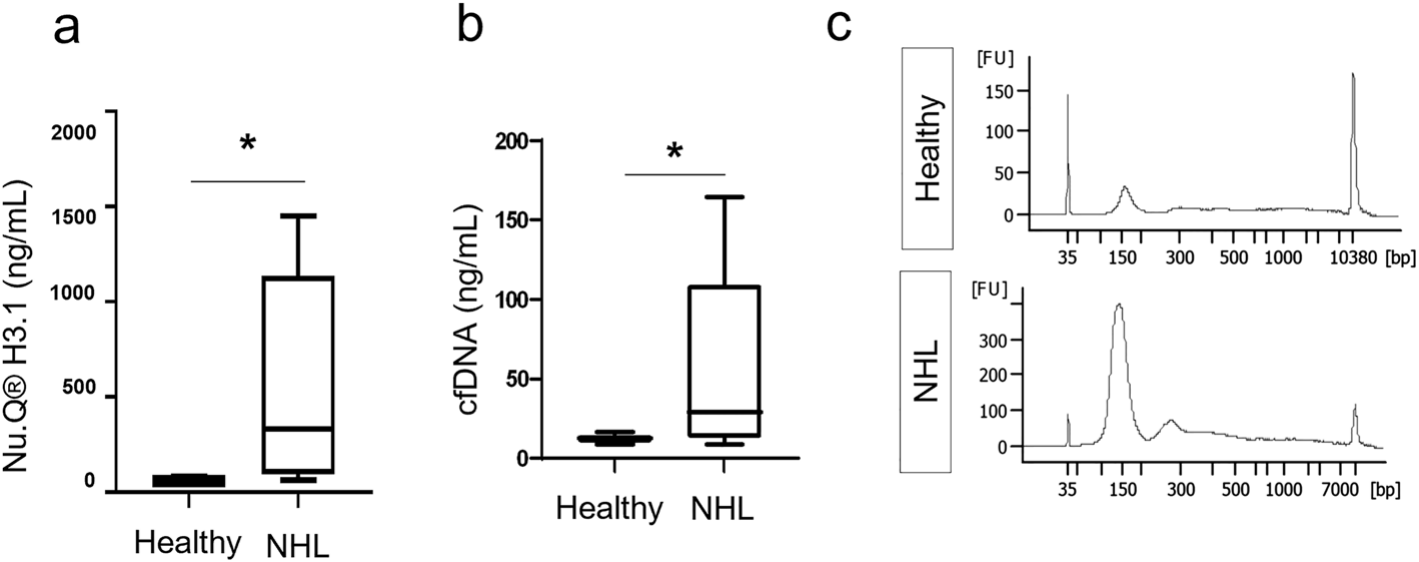
Levels of H3.1-nucleosomes and cfDNA in NHL and in healthy samples. (**a**) Significant increase of the H3.1-nucleosome concentration (ng/mL) in NHL samples (n = 9) compared to healthy samples (n = 5) (*p < 0.05). (**b**) Significant higher levels of cfDNA (ng/mL) in plasma from NHL samples (n = 7) compared to healthy samples (n = 5) (*p < 0.05). Box plots show the median and the 25th and 75th percentiles; the whiskers indicate the min and the max values. *p* values were determined by Mann–Whitney. (**c**) cfDNA Fragment size distribution of a representative healthy sample compared to NHL sample using Agilent 2100 Bioanalyzer. Additional electropherograms are shown in Supplementary Figure 1.

Levels of H3.1-nucleosomes and cfDNA were measured in plasma samples from NHL patients and healthy donors. Results of the Nu.Q® H3.1 immunoassay showed significantly higher concentrations of circulating H3.1-nucleosomes in NHL samples (mean: 582.3 ng/mL ± 564.4, n = 9 patients) compared to healthy samples (mean: 56.18 ng/mL ± 29.28, n = 5 donors, *p* = 0.012) (Fig. 1a). As nucleosomes are composed of DNA wrapped around a core octamer of histone proteins, we next assessed the total level of cfDNA from leftover plasma of the same NHL patients and healthy donors. The Qubit DNA quantification also showed an increase of cfDNA levels in NHL samples (mean = 53.68 ± 59.73 ng/mL, n = 7) versus healthy samples (mean = 12.32 ± 2.823 ng/mL; n = 5; *p* = 0.037) (Fig. 1b). As anticipated, there was a significant strong association between cfDNA concentration and the amount of H3.1-nucleosomes (Spearman’s correlation coefficient r: 0.762, *p* = 0.006). Fragment size analysis of the cfDNA extracted from NHL samples showed that cfDNA size in NHL patients was centered around 157.7 bp ± 7.653 bp (range 147 bp—168 bp) corresponding to the length of DNA wrapped around a single nucleosome with some dinucleosomes DNA length (~ 280 bp) present (Fig. 1c and Supplementary Figure 1). Similarly, the cfDNA lengths from healthy samples correspond to the mononucleosome size but with a slightly longer associated-DNA (range 160–175 bp). These results confirm that cfDNA is mainly bound to H3.1-nucleosomes in NHL samples. Altogether, these results show high levels of H3.1-nucleosomes, as well as cfDNA, in the K2-EDTA plasma samples from NHL patients.

### Identification of differential levels of histone PTMs in NHL samples by Nu.Q® Capture-Mass Spectrometry

**Figure 2:**
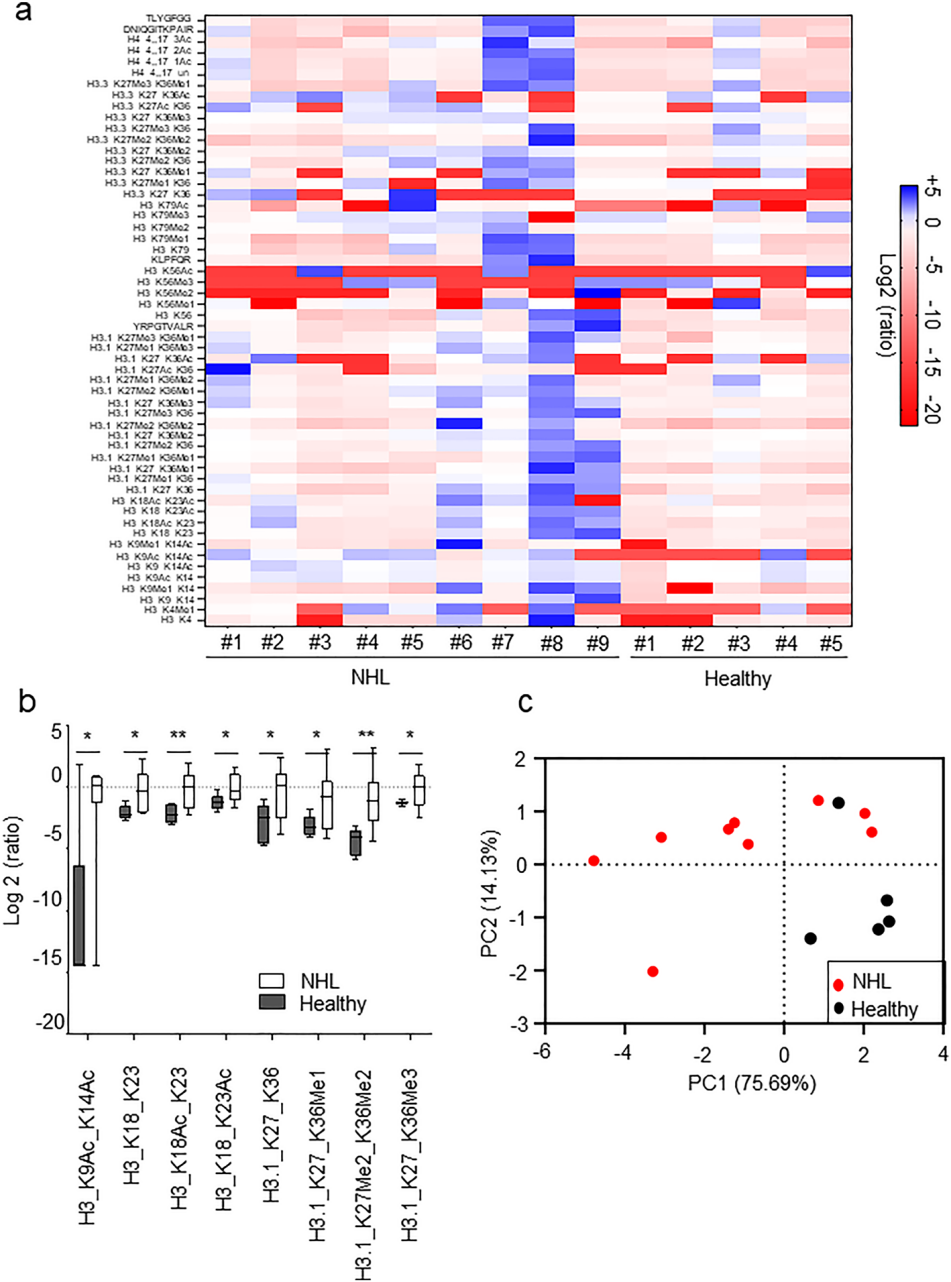
Nu.Q® Capture—MS allows the epigenetic profiles of circulating nucleosomes of NHL patients. (**a**) Heat map showing 56 histone PTMs peptides identified on captured circulating nucleosomes from NHL samples (n = 9 labelled from #1 to #9) and healthy samples (n = 5 labelled from #1 to #5). Data are shown as log2 ratio of histone PTMs levels. (**b**) Box plot showing the abundance of histone peptides detected by Nu.Q® Capture-MS in plasma sample from healthy donors (n = 5) compared to NHL patients (n = 9). *p values* were determined by ANOVA Test (*p < 0.05; **p < 0.01) and results are expressed as log2 (ratio) of histone PTMs levels. (**c**) Principal component analysis. Each point represents a sample. The variance explanation of the principal component (PC) is expressed in % under the related axis.

Comprehensive histone PTMs profiling of circulating nucleosomes in plasma from NHL patients was performed using a previously established Nu.Q® Capture—MS protocol^23^. Nucleosome quantification showed a mean depletion of 89.6% ± 6.9% of the neat sample after Nu.Q® Capture confirming efficient nucleosome immunoprecipitation and nucleosomes enrichment on beads (Supplementary Figure 2). 56 different histones peptides, mainly localized in the histone H3 and on the histone H4 N-terminal tails were identified using Skyline software (Fig. 2a). Statistical analysis of normalized peptide ratio highlighted 8 histone proteoforms that were differentially represented in plasma from NHL patients compared to healthy donors (*p* < 0.05) (Fig. 2b). Notably, we observed higher levels of acetylated peptides (H3K9Ac, H3K14Ac, H3K18Ac and H3K23Ac) as well as elevated methylated histone H3 peptides (H3K36Me1/2/3 and H3K27Me2). To further examine the relationships between the 8 distinct histone PTMs significantly elevated in NHL and the health status (i.e. NHL vs healthy), we performed a principal component analysis (PCA). The variance explanation chart indicated that the two first principal components (PC) were responsible for about 89.82% of the variance (PC1: 75.69% and PC2: 14.13%), separating most of the healthy donors from the NHL patients (Fig. 2c). Loading plots highlighted that the PC1 is mainly influenced by H3K36Me1 (principal component coefficient: − 0.960) and H3K18Ac (principal component coefficient: − 0.962) while the H3K9Ac/H3K14Ac (principal component coefficient: 0.933) are the main contributor of the PC2 (Supplementary Figure 3). Altogether, these results suggest that Nu.Q® Capture—MS protocol allows the histone PTMs pattern identification that could be used to separate NHL from healthy samples.

### Confirmation of elevated histone PTMs levels in NHL patients by Nu.Q® immunoassay

**Figure 3:**
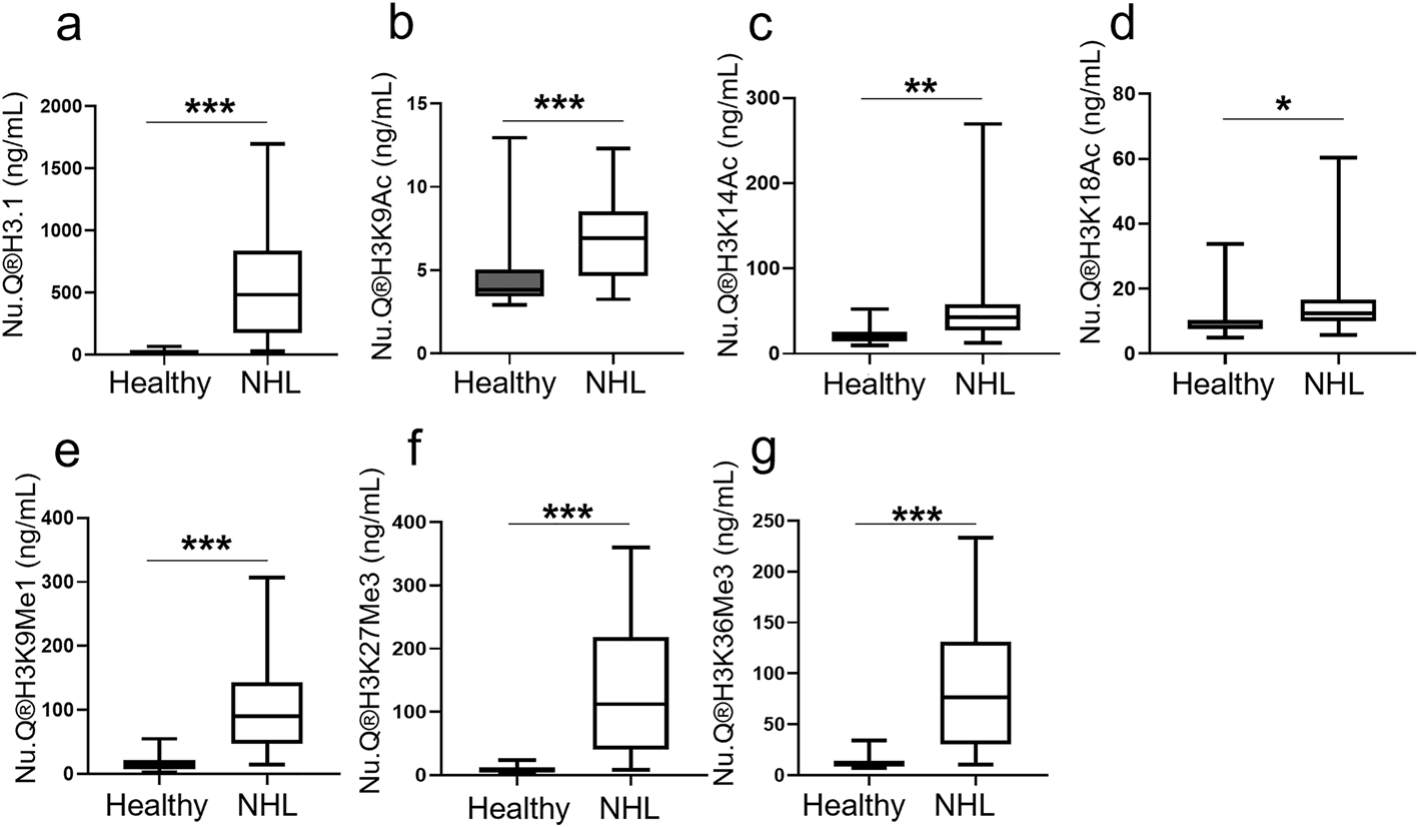
H3.1-nucleosomes and nucleosomes containing histone PTMs identified by Mass spectrometry in healthy versus NHL samples. Box plot showing quantifications by immunoassays of modified circulating H3.1- (***p < 0.001) (**a**), H3K9Ac- (***p < 0.001) (**b**), H3K14Ac- (**p = 0.001) (**c**), H3K18Ac- (*p = 0.022) (**d**), H3K9Me1- (***p < 0.001) (**e**), H3K27Me3- (***p < 0.001) (**f**), H3K36Me3- (***p < 0.001) nucleosomes (**g**) from NHL samples (n = 24) compared to healthy samples (n = 34). *p* values were determined by Mann–Whitney.

The capability of the MS-identified histone PTMs to differentiate between NHL and healthy samples was further tested in an independent cohort (n = 24 NHL patients and n = 35 healthy donors) using available quantitative Nu.Q® immunoassays targeting 7 of the 8 differentially expressed histone PTMs identified by MS (Fig. 3). The level of H3.1-nucleosomes was drastically increased in NHL samples compared to healthy samples. The median was 483.6 ng/mL in NHL samples (range 28.8–1699 ng/mL, n = 22) versus 12.11 ng/mL for healthy samples (range 4.3–67.47 ng/mL, n = 35) (*p* < 0.001; Fig. 3a). Moreover, NHL patients had significantly higher levels of nucleosome with histone H3 acetylation (median H3K9Ac = 6.93 ng/mL; H3K14Ac = 42.99 ng/mL; H3K18Ac = 12.40 ng/mL; Fig. 3b–d) and methylation (median H3K9Me1 = 90.03 ng/mL; H3K27Me3 = 112.09 ng/mL; H3K36Me3 = 76.27 ng/mL; Fig. 3e–g) when compared to healthy controls medians (median H3K9Ac = 3.83 ng/mL; H3K14Ac = 18.67 ng/mL; H3K18Ac = 8.15 ng/mL; H3K9Me1 = 13.97 ng/mL; H3K27Me3 = 8.79 ng/mL; H3K36Me3 = 10.14 ng/mL; *p* < 0.05) (Supplementary Table 3). Overall, these immunoassays results confirmed the elevation of H3.1-circulating nucleosomes and the nucleosome containing the histone PTMs discovered by mass spectrometry.

### Nucleosome concentrations are altered with response to chemotherapy

**Figure 4:**
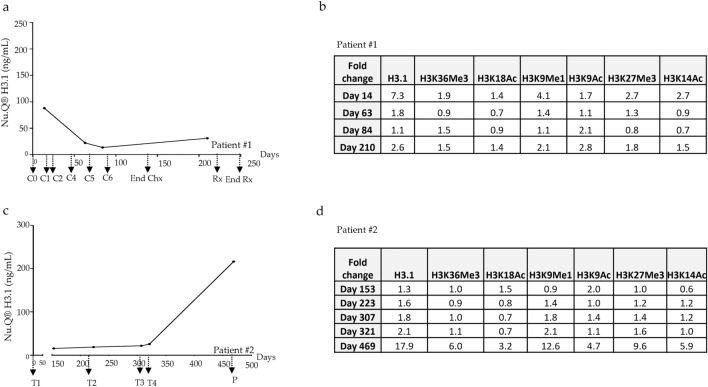
Longitudinal assessment of nucleosome concentrations in NHL samples (n = 2) throughout the course of treatment. (**a, c**) Temporal distribution of H3.1-nucleosomes level expressed in ng/mL at different timepoints. (**b, d**) Fold change levels of specific PTMs-nucleosomes (H3.1-, H3K36Me3-, H3K18Ac-, H3K9Me1-, H3K9Ac-, H3K27Me3- and H3K14Ac-nucleosomes) compared to the samples. C1 to C6: chemotherapy cycle from 1 to 6; End of Chx: end of chemotherapy, , T1 to T4: treatment from 1 to 4, P: progression, Rx: radiation, End Rx: End of the radiations.

As we found that levels of circulating H3.1-nucleosomes containing histones H3 PTMs were upregulated in patients with NHL, we then evaluated their temporal distribution as well as the variation related to the median level of the reference healthy samples (hereafter referred as fold-change) during the treatment course (Fig. 4 and Supplementary Table 4). In patient #1, at the first measurement after the first cycle of chemotherapy (C1 at day 14), the highest fold-change among all the nucleosome marks analyzed was observed for the H3.1-nucleosome level with a 7.3-fold change and a concentration that reached 87.98 ng/mL (Fig. 4a, b). Then, the H3.1-nucleosome levels decreased drastically during the first cycles of chemotherapy to a level of 22.1 ng/mL after day 63 (C5) close to the healthy reference level (1.8-fold-change). At day 210, we observed a very slight increase up to the final treatment time point (30.98 ng/mL and 2.6-fold change) (Fig. 4a). Similarly, the H3K9Me1- and H3K14Ac-nucleosome levels all fell after the administration of the drugs from a fold change of 4.1 to 1.1 for H3K9Me1-nucleosome and from 2.7 to 0.7 for H3K14Ac-nucleosomes. While the level of H3K36Me3-, H3K27Me3- and H3K18Ac-nucleosomes remained close to the reference level of the healthy samples (fold change < 2), we noticed a gradual increase of H3K9Ac-nucleosome (from a healthy related fold change of 1.7 to a higher fold change of 2.8) during the treatment. In patient #2, pre-treatment level of circulating nucleosomes was not available until day 153. During treatment, the level of H3.1-nucleosomes was maintained from day 153 to day 321 (H3.1: 20.5 ± 4.3 ng/mL) followed by an increase on day 469 (H3.1: 216.37 ng/mL). At this date the patient was defined as a non-responder to therapy and exhibited a progressive NHL disease (Fig. 4c). Similarly to H3.1-nucleosome level, the fold change levels of all modified nucleosomes (H3K9Ac-, H3K14Ac-, H3K18Ac-, H3K9Me1-, H3K27Me3- and H3K36Me3-nucleosomes) also increased (fold change between 3.2 and 12.6) in levels on day 469 (Fig. 4d). Interestingly, at day 321, before the diagnosis of the disease progression, we already observed an elevation of H3.1- and H3K9Me1-nucleosome level (fold change: 2.1). Altogether, these results show high levels of H3.1-nucleosomes and some histone-PTMs at diagnosis (patient#1) or progressive NHL disease (patient#2).

## Discussion

Liquid biopsies offer a promising diagnosis tool for cancer detection as it is noninvasive and easily repeatable over time compared to tissue biopsy. Recently, several papers have suggested that cfDNA increases in NHL blood compared with the healthy control group^17,19^. In this study, we describe high levels of circulating H3.1-nucleosomes in human plasma from NHL patients compared to healthy donors^25,26^. This level of H3.1-nucleosome was correlated with an elevated concentration of cfDNA which was consistent with DNA size corresponding to the length of DNA wrapped around the nucleosomes with the presence or absence of linker DNA. Several studies have also demonstrated that circulating H3.1-nucleosomes can be elevated in many solid cancer patients including lung cancer, breast, prostate colorectal cancer in comparison to healthy donors, particularly in cases of late cancer stages^29,30^. Since nucleosomes can be released by cancer cells into the blood, they contain the epigenetic signature of the tumor and the detection and quantification of both nucleosomes and their associated PTMs looks primising cancer biomarkers^31^. For instance, in colorectal cancer, the H3K27Me2, H3K27Ac, and H2A1R3Cit histone-PTMs present on circulating nucleosome reflects the epigenetic signature of the tumor^23^. In lung cancer, significantly elevated concentrations of H3K27Me3 nucleosomes were found in plasmas at the diagnosis, and during the follow-up, of NSCLC (non-small-cell lung cancer) patients, compared to healthy donors^32^.Mass spectrometry has emerged as a powerful tool for the unbiased and quantitative evaluation of proteins and their posttranslational modifications^24^. In lymphoma, most of the current available studies using mass spectrometry characterize the PTMs on the global phosphoproteome of proteins^33–35^. In addition, mass spectrometry and/or immuno-based analysis have identified altered epigenetic enzymes such as the methyltransferase^36^, acetyltransferases^9,10^, histones deacetylase^37^ and demethylases^38^ in lymphoma^39–41^ without, however, extensive characterization of circulating histone PTMs pattern. Recently, we described a new nucleosome-enrichment method followed by mass spectrometry to decipher the epigenetic pattern on circulating nucleosome in plasma of colorectal cancer patients^23^. This protocol used a validated anti-H3.1 antibody targeting specifically the Histone H3.1 variant. Because the N-terminus of the histone H3 has been extensively described to exhibit post-translational modifications that influence cellular processes and tumorigenesis, we decided to analyze predominantly the PTMs present on this histone. Moreover, the interest of targeting the H3.1- nucleosome is also linked to its proximity to the value of total nucleosomes described in the literature as increased in cancer^30,42^. We have already confirmed by immunoassay, a higher level of circulating H3.1- nucleosomes in hematological malignancy (poster E20078, ASCO 2020), and in canine cancer^43^. Consistently, in the present study, we demonstrated that the level of circulating H3.1 nucleosome is increased in the NHL plasma samples. Then, we have applied the Nu.Q®Capture-mass spectromety protocol to a liquid tumor in a pilot study composed by NHL patients and healthy donors and have identified for the first time an epigenetic pattern of circulating nucleosomes dysregulated in NHL patients in comparison with the healthy donors. Our analysis demonstrated increased levels of nucleosomes containing acetylated and/or methylated lysine on histone H3 in plasma from NHL patient. Furthermore, these elevated histone PTM patterns were confirmed in an independent cohort using immunoassays targeting H3.1-, H3K9Me1-, H3K27Me3-, H3K36Me3-, H3K9Ac-, H3K14Ac-, H3K18Ac-nucleosomes in NHL samples in comparison with healthy samples. During the statistical analysis, we observed that samples NHL#8 and #9 exhibit a higher level of PTM dysregulation. The level of H3.1-nucleosomes in these samples was particularly elevated (> 1350 ng/mL) potentially indicating a more aggressive or progressive stage of disease or comorbidities contributing to the elevated nucleosome levels. Nevertheless, principal component analysis of the histone PTMs significantly upregulated in NHL blood could separate NHL samples from most healthy samples, suggesting that the identified histone PTMs could be promising diagnostic biomarkers. In line with our results, it has been recently demonstrated that histone methyl transferases such as EZH2 are misregulated or mutated in NHL disease leading to an aberrant methylation of H3K27, and silencing of tumor suppressors contributing to disease progression^7,44^. The epigenetic regulation of chromatin is a dynamic process that allows a focal architectural remodeling of the genomic DNA that shifts from a compacted chromatin to a relaxed, activated state. This activation is associated with a hyperacetylation of histone H3 and H4 and the presence of H3K4Me3^7,45^. Consistently, in this study, we observed a significant increase of acetylation of the histone H3 histones in NHL (H3K9Ac, H3K14Ac, H3K18Ac and H3K23Ac) suggesting an active transcription state. Moreover, H3K18Ac marks are known to play a major role in disease progression of several cancers^46,47^. We hypothesized that it could act similarly in NHL cancer. Interestingly, H3K36Me3 has not yet been extensively studied in NHL patients but an accumulation of this mark mediated by SETD2 or NSD2 has been associated with progression of acute myeloid leukemia^48,49^. We can speculate that the same phenomenon could be observed in lymphoma.

After identification of a dysregulated epigenetic pattern in NHL disease, we looked at it to evaluate the therapeutic response and monitoring of tumor progression of two patients under chemotherapy. Traditionally, imagery including the positron emission tomography is widely used for the treatment evaluation of NHL^50^. The current absence of valuable noninvasive methods for predicting and/or detecting therapeutic response of NHL tumors limits patient care. Here, we report the benefits of circulating nucleosomes as non-invasive biomarkers for NHL progression and monitoring response to treatment. The first patient exhibited a rapid decrease in H3.1-nucleosome levels as well as the H3K9Me1- and H3K14Ac-nucleosomes levels after one cycle of therapy supporting a good response to the chemotherapy. In the second patient, a substantial increase in overall nucleosome levels as well as increases in all measured PTMs was seen at the final time point at the last measurement. Clinical records indicated this patient was “suffering of a progressive disease” at this time point. In line with what has been described in rodents with Hela xenografts^51^ as well as in humans^51–53^, our data suggest that the concentration of circulating H3.1-nucleosome could be used as an indicator of a progressive NHL cancer. Before the diagnosis of the disease progression, we had already observed an elevation of H3.1- and H3K9Me1-nucleosome level (patient #2) opening the door to use circulating nucleosome and associated histone PTMs as early detectors of treatment failure/progressive disease. Since cancer treatment can modulate epigenetic modifier activity^54^ or HDAC inhibitor^13^ the histone PTMs of interest could have an added value to monitor the treatment response. Nevertheless, further work investigating the links between circulating nucleosome PTMs, and treatment monitoring are required to provide clinically meaningful data of these new potential biomarkers. Indeed, since all samples used in this study were collected retrospectively, we have faced limitations including (1) the limited volume of samples available which did not allow all of the experiments to be carried out on all samples; (2) the access to the clinical data is limited which does not allow in-depth clinical analysis of the results, (3) the low number of samples processed by MS and the absence of stratification based on the tumor stages or subtypes could mask subtle variations in epigenetic marks.

In summary, our work emphasizes the crucial roles of the epigenetic marks present on circulating nucleosomes to detect and monitor diseases such as lymphoma. However, the clinical data presented here have some limitations. Firstly, the low number of samples processed by MS and the absence of stratification based on the tumor stages or subtypes could mask subtle variations in epigenetic marks. Therefore, further clinical studies with larger cohorts should be carried out to validate the present results and/or highlight other histone PTMs not yet investigated. Secondly, our method of enrichment (Nu.Q® capture) is specific to H3.1-nucleosome but we identified H3.3 related peptides by mass spectrometry. This unexpected observation has been already observed in colorectal cancer^23^ and could be due to either the presence of asymmetric H3.1-H3.3 nucleosomes^55^ or the presence of di- and poly-nucleosomes in which the H3.1-nucleosome could be associated with another H3.3-nucleosome. Distinct types of histone modifications are known, amongst which acetylation, methylation, phosphorylation, citrullination and ubiquitination are the best studied and the most important in terms of the regulation of the transcription activity. While acetylation and methylation are well identified in the present study, the other PTMs were not detected perhaps due to their low abundance in free nucleosomes in plasma or due to MS parameters. The variations of H4K20Me3, H3K9Me3, and H3K14Ac levels are intensively described to be the hallmark of cancer^24,51–53^. However, in the present study, we mainly focused on the PTMs associated with the histone H3 and the acetylation state of the only first part of histone H4 peptide (H4_4…17). Therefore, it could be interesting to complete this PTMs characterization in circulating nucleosomes by targeting these other PTMs.

## Conclusions

In conclusion, our data confirmed reported elevated cfDNA in NHL and also showed, for the first time, a strong correlation of cfDNA with H3.1-nucleosome levels. One of the advantages of nucleosome levels measurement compared to DNA measurement is that it does not require any extraction, plasma can be directly analyzed which makes its use easier. Finally, we demonstrated the potential use of nucleosome levels as new liquid biopsy biomarkers to evaluate therapeutic response and for monitoring tumor progression. Plasma nucleosome concentrations and a subset of histone PTMs reflect the clinical course of disease in the two cases presented whilst the magnitude of the change in nucleosome levels at diagnosis of relapse points to potentially much earlier diagnosis of treatment failure.

### Supplementary Information

Supplementary Figure 1.

Supplementary Figure 2.

Supplementary Figure 3.

Supplementary Legends.

Supplementary Tables.

## Data Availability

The datasets used and/or analyzed during the current study are available from the corresponding author on reasonable request.

## Abbreviations

BPH: Benign prostatic hyperplasia
cfDNA: Circulating cell-free DNA
C1 to C6: Chemotherapy cycle from 1 to 6
CAD: Coronary artery disease
Chx: End of chemotherapy
DLBCL: Diffuse large B cell lymphoma
GCB: Germinal-center-B-cell-like
HATs: Histone acetyltransferases
HDACs: Histone deacetylases
LC–MS/MS: Tandem mass spectrometry
MS: Mass spectrometry
NHL: Non-Hodgkin lymphoma
P: Progression disease
PC: Principal component
PCA: Principal component analysis
PTMs: Post-translational modifications
RLU: Relative light unit
Rx: Radiation therapy
T1 to T4: Treatment from 1 to 4
