# Supplementary figures and images for: Epigenetic profiles of elevated cell free circulating H3.1 nucleosomes as potential biomarkers for non-Hodgkin lymphoma
Source: Sci Rep. 2023 Sep 28;13:16335. doi: 10.1038/s41598-023-43520-0 (PMC10539380; doi:10.1038/s41598-023-43520-0)

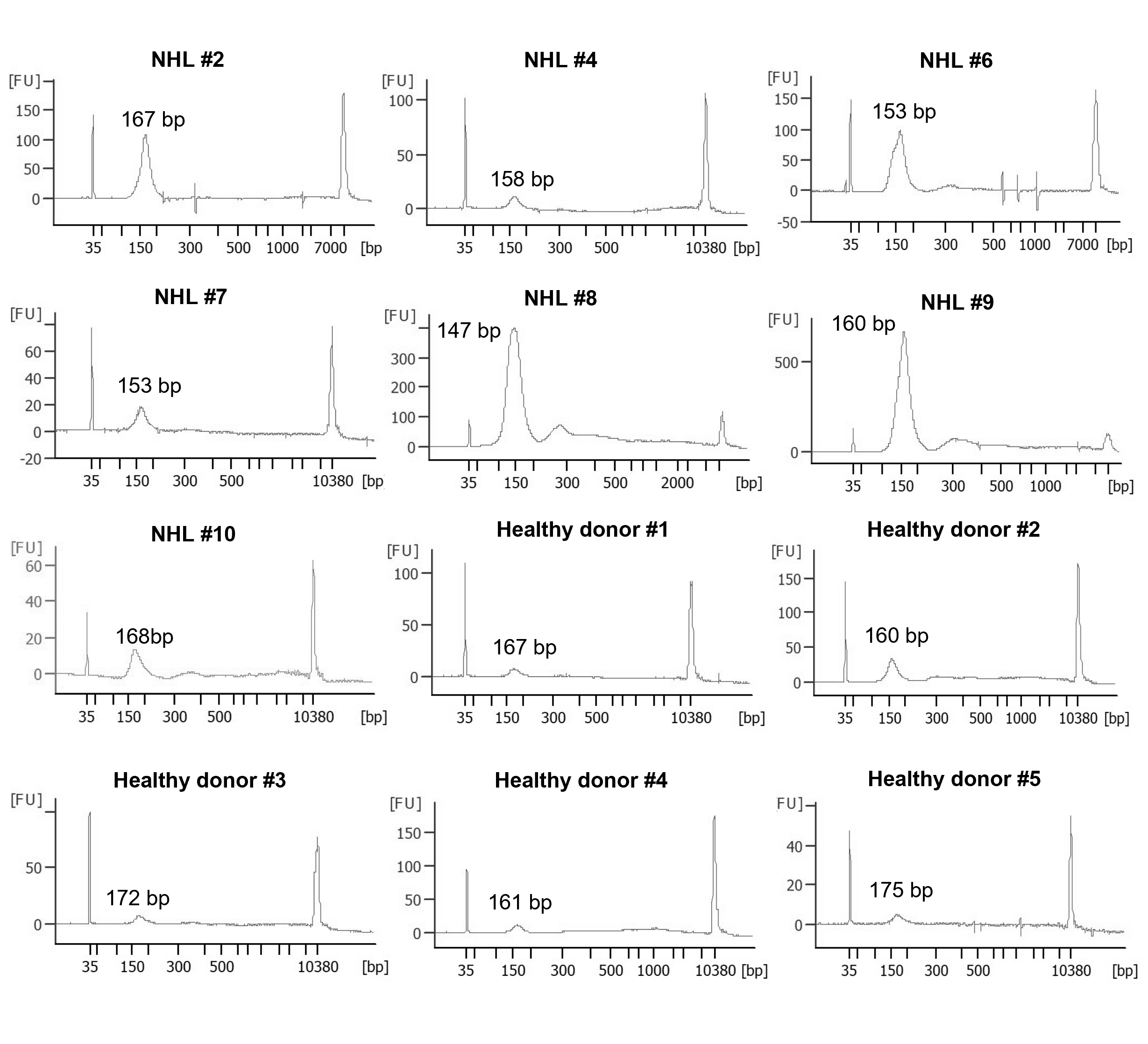

Supplement: Supplementary file 1 — Supplementary Figure 1. [file 41598_2023_43520_MOESM1_ESM.tif]

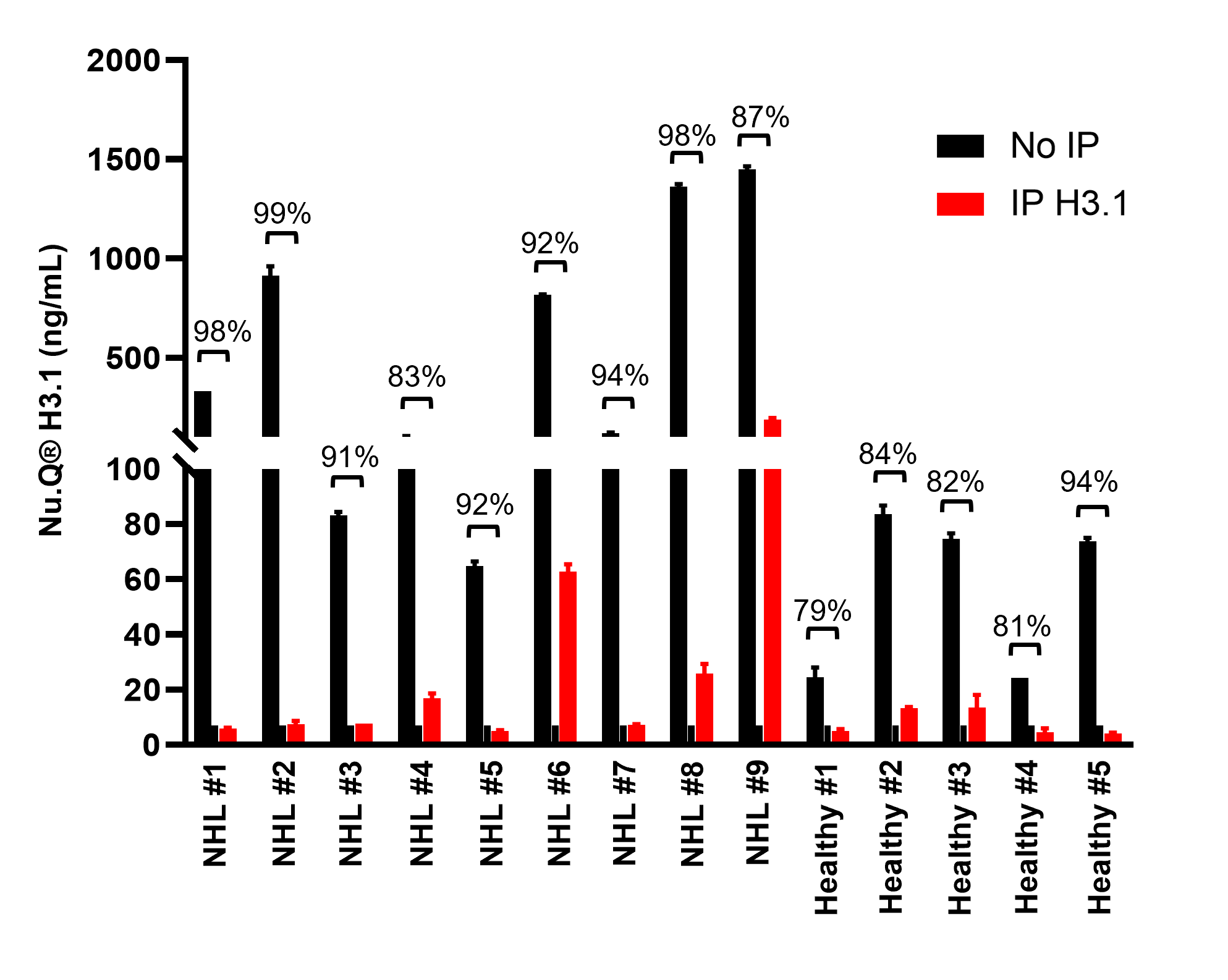

Supplement: Supplementary file 2 — Supplementary Figure 2. [file 41598_2023_43520_MOESM2_ESM.tif]

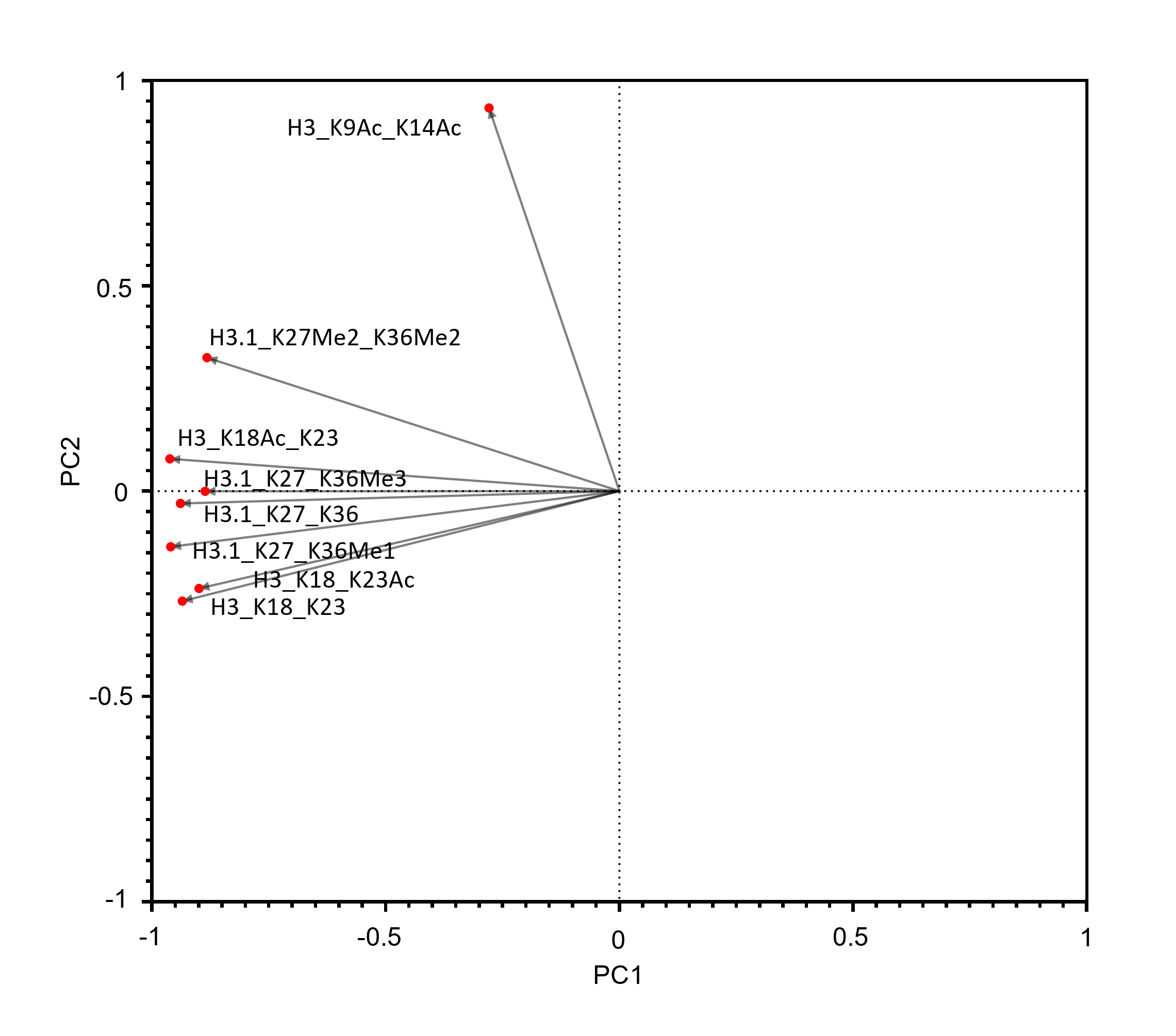

Supplement: Supplementary file 3 — Supplementary Figure 3. [file 41598_2023_43520_MOESM3_ESM.tif]
