# Supplementary material for: Epigenetic profiles of elevated cell free circulating H3.1 nucleosomes as potential biomarkers for non-Hodgkin lymphoma
Source: Sci Rep. 2023 Sep 28;13:16335. doi: 10.1038/s41598-023-43520-0 (PMC10539380; doi:10.1038/s41598-023-43520-0)
Supplement: Supplementary file 4 — Supplementary Legends. [file 41598_2023_43520_MOESM4_ESM.docx]

**Supplementary information**

**Supplementary Figure 1. cfDNA fragment size distribution of healthy donors compared to NHL patients using Agilent 2100 Bioanalyzer**. The peaks at 35 and 10,000 bp correspond to the two internal size markers. The relative fluorescence (FU, Y-axis) of these markers is used to calculate the size of the cfDNA samples (bp, x-axis). The number recorded on the electropherograms is automatically provided by the Agilent 2100 Bioanalyzer profiles and corresponds to the DNA length (bp) of the max pic sizing. The samples are labelled from #1 to #10 and the associated number is kept across the different experiments.

**Supplementary Figure 2**. **Nu.Q® Capture Mass spectrometry allows the depletion of H3.1- positive nucleosomes in plasma K2EDTA samples**. Nu.Q® H3.1 immunoassay results showing a mean depletion of 89.6% ± 6.9% of nucleosomes after Nu.Q® Capture protocol (red bar) in comparison to the level present in the initial plasma sample (black bar). All data are presented as mean ± SD and results are expressed in ng/mL. The percentage of depletion was indicated for all samples (n=5 healthy donors and n=9 NHL).

**Supplementary Figure 3**. **Principal component analysis : The** **loadings plot.** The loadings plot shows the variables (histone-PTMs) that contributed most to the variance along the first and second principal components (PC1 and PC2).

**Supplementary Table 1.** **Clinical samples collection / cohort information.** Table showing the age, sexe, clinical diagnosis and medical history for each patient. NHL: Non-Hodgkin lymphoma, DLBCL: Diffuse large B-cell lymphoma, GCB: Germinal-center-B-cell-like, CAD: Coronary artery disease, BPH: Benign prostatic hyperplasia.

**Supplementary Table 2**. **Clinical samples collection / cohort information**. Table showing the clinical state, age and sexe for each patient. DLBCL: Diffuse large B-cell lymphoma.

**Supplementary Table 3. Post-translational modifications of the histone H3 identified by mass spectrometry in NHL and healthy samples**. Table showing the comparison levels of H3.1, H3K9Ac, H3K14Ac, H3K18Ac, H3K9Me1, H3K27Me3 and H3K36Me3 between the NHL and the healthy samples. Median, Mean, SD value and Range are expressed as ng/mL.

**Supplementary Table 4. Nucleosome concentrations are altered with response to therapy**. Table showing the level of H3.1-, H3K36Me3-, H3K18Ac-, H3K9Me1-, H3K9Ac-, H3K27Me3- and H3K14Ac-nucleosomes expressed as ng/mL for patient #1 and patient #2 throughout the course of their treatment. C1 to C6 : Chemotherapy cycle from 1 to 6; End of Chx: end of chemotherapy, T1 to T4: treatment from 1 to 4.
