# Supplementary material for: Epigenetic profiles of elevated cell free circulating H3.1 nucleosomes as potential biomarkers for non-Hodgkin lymphoma
Source: Sci Rep. 2023 Sep 28;13:16335. doi: 10.1038/s41598-023-43520-0 (PMC10539380; doi:10.1038/s41598-023-43520-0)
Supplement: Supplementary file 5 — Supplementary Tables. [file 41598_2023_43520_MOESM5_ESM.doc]

**Supplementary** Table 1

|  | **Sample ID** | **Age** | **Sexe** | **Clinical diagnosis** | **Medical history** |
| --- | --- | --- | --- | --- | --- |
| **NHL** | #1 | 61 | Female | DLBCL, non-GCB - type | Hypertension, obesity |
|  | #2 | 39 | Male | Follicular lymphoma, type 1-2 | No |
|  | #3 | 81 | Male | DLBCL | Diabetes type 2; CAD; Hypertension; atherosclerosis; obesity |
|  | #4 | 70 | Male | DLBCL | Hypertension; BPH |
|  | #5 | 66 | Male | DLBCL (non-GCB - type) | Chronic eosophagitis |
|  | #6 | 69 | Female | Follicular lymphoma, type 3A | Hypertension; obesity |
|  | #7 | 36 | Male | DLBCL | No |
|  | #8 | 66 | Male | Follicular lymphoma, type 3A | No |
|  | #9 | 66 | Female | DLBCL (non GCB - type) | CAD; hypertension; atherosclerosis |
| **Healthy donors** | #1 | 72 | Female | NA | History of colorectal polyps |
|  | #2 | 59 | Male | NA | History of colorectal polyps |
|  | #3 | 69 | Male | NA | History of colorectal polyps |
|  | #4 | 57 | Male | NA | History of colorectal polyps |
|  | #5 | 71 | Female | NA | History of colorectal polyps |

**Supplementary Table 2**

| **Sample ID** | **Clinical state** | **Age** | **Sexe** |
| --- | --- | --- | --- |
| #1 | Healthy donor | 19 | Female |
| #2 | Healthy donor | 55 | Female |
| #3 | Healthy donor | 36 | Female |
| #4 | Healthy donor | 42 | Female |
| #5 | Healthy donor | 53 | Male |
| #6 | Healthy donor | 43 | Male |
| #7 | Healthy donor | 64 | Male |
| #8 | Healthy donor | 18 | Female |
| #9 | Healthy donor | 36 | Male |
| #10 | Healthy donor | 63 | Male |
| #11 | Healthy donor | 70 | Female |
| #12 | Healthy donor | 27 | Female |
| #13 | Healthy donor | 23 | Female |
| #14 | Healthy donor | 56 | Male |
| #15 | Healthy donor | 63 | Male |
| #16 | Healthy donor | 54 | Male |
| #17 | Healthy donor | 23 | Female |
| #18 | Healthy donor | 43 | Female |
| #19 | Healthy donor | 57 | Female |
| #20 | Healthy donor | 45 | Male |
| #21 | Healthy donor | 48 | Female |
| #22 | Healthy donor | 65 | Female |
| #23 | Healthy donor | 54 | Male |
| #24 | Healthy donor | 22 | Male |
| #25 | Healthy donor | 27 | Female |
| #26 | Healthy donor | 26 | Female |
| #27 | Healthy donor | 38 | Female |
| #28 | Healthy donor | 21 | Female |
| #29 | Healthy donor | 37 | Female |
| #30 | Healthy donor | 18 | Female |
| #31 | Healthy donor | 70 | Male |
| #32 | Healthy donor | 51 | Female |
| #33 | Healthy donor | 49 | Female |
| #34 | Healthy donor | 27 | Female |
| #35 | Healthy donor | 42 | Female |
| #36 | DLBCL, Stage IIb; Grade G2 | 41 | Female |
| #37 | DLBCL, Stage IIIb; Grade G3 | 60 | Male |
| #38 | DLBCL, Stage IIb; Grade G3 | 50 | Male |
| #39 | Hodgkin Lymphoma, Stage IIa; Grade G2 | 39 | Female |
| #40 | DLBCL, Stage II; Grade G3 | 56 | Female |
| #41 | DLBCL, Stage IIa; Grade G3 | 54 | Female |
| #42 | DLBCL, Stage IVb; Grade G3 | 38 | Male |
| #43 | DLBCL, Stage II; Grade G3 | 62 | Male |
| #44 | DLBCL, Stage IVa; Grade G3 | 54 | Male |
| #45 | DLBCL, Stage IIIb; Grade G3 | 59 | Male |
| #46 | DLBCL, Stage IIa; Grade G3 | 68 | Male |
| #47 | DLBCL, Stage IIa; Grade G3 | 46 | Male |
| #48 | DLBCL, Stage IIb; Grade G3 | 78 | Male |
| #49 | DLBCL, Stage IVb; Grade G3 | 56 | Male |
| #50 | DLBCL, Stage IV; Grade G3 | 53 | Male |
| #51 | DLBCL, Stage IIa; Grade G3 | 65 | Female |
| #52 | Hodgkin Lymphoma, Stage IVb; Grade G3 | 58 | Female |
| #53 | Hodgkin Lymphoma, Stage IIIb; Grade G3 | 54 | Female |
| #54 | DLBCL, Stage IVa; Grade G4 | 62 | Male |
| #55 | DLBCL, Stage IVb; Grade G3 | 72 | Male |
| #56 | DLBCL, Stage IIb; Grade G3 | 69 | Male |
| #57 | DLBCL, Stage IVb; Grade G3 | 58 | Male |
| #58 | DLBCL, Stage IIIb; Grade G3 | 78 | Male |
| #59 | Hodgkin Lymphoma, Stage IIb; Grade G2 | 48 | Female |

**Supplementary Table** 3

|  | **Histone PTMs** | **Number of samples** | **Median** | **Mean** | **SD** | **Range** |
| --- | --- | --- | --- | --- | --- | --- |
| **NHL** | H3.1 | 22 | 483.6 | 578.5 | 505.78 | 28.80 – 1699.46 |
|  | H3K9Ac | 24 | 6.93 | 7.008 | 2.44 | 3.26 – 12.29 |
|  | H3K14Ac | 24 | 42.99 | 51,91 | 51.03 | 12.92 – 269.73 |
|  | H3K18Ac | 24 | 12.4 | 14,9 | 10.57 | 5.79 – 60.43 |
|  | H3K9Me1 | 24 | 90.03 | 100.3 | 66.43 | 14.26 – 306.39 |
|  | H3K27Me3 | 24 | 112.09 | 130.5 | 102.98 | 7.98 – 360.22 |
|  | H3K36Me3 | 24 | 76.27 | 85.59 | 62.91 | 10.17 – 233.24 |
| **Healthy donors** | H3.1 | 35 | 12.11 | 14.13 | 11.14 | 4.30 – 67.46 |
|  | H3K9Ac | 34 | 3.83 | 4.575 | 2.02 | 2.93 – 12.96 |
|  | H3K14Ac | 34 | 18.67 | 21.29 | 9.53 | 9.75 – 52.40 |
|  | H3K18Ac | 33 | 8.15 | 9.873 | 5.36 | 5.03 – 33.81 |
|  | H3K9Me1 | 34 | 13.97 | 16.63 | 12.29 | 2.50 – 54.99 |
|  | H3K27Me3 | 34 | 8.79 | 8.696 | 4.39 | 2.99 – 23.85 |
|  | H3K36Me3 | 34 | 10.14 | 12.13 | 5.91 | 7.18 – 34.02 |

**Supplementary Table 4**

|  | **Nu.Q® H3.1** | **Nu.Q® H3K36Me3** | **Nu.Q® H3K18Ac** | **Nu.Q® H3K9Me1** | **Nu.Q® H3K9Ac** | **Nu.Q® H3K27Me3** | **Nu.Q® H3K14Ac** | **Time point (collection)** | **Cycle Tx** | **Days** | **Treatment** |
| --- | --- | --- | --- | --- | --- | --- | --- | --- | --- | --- | --- |
| **Patient #1** | - | - | - | - | - | - | - | - | C0 | 0 | No previous treatment |
|  | - | - | - | - | - | - | - | - | C1 | 1 | R-CHOP |
|  | 87.98 | 19.33 | 11.30 | 56.78 | 6.68 | 23.90 | 49.53 | 1 |  | 14 | - |
|  | - | - | - | - | - | - | - | - | C2 | 21 | Elitek® or R-CHOP |
|  | - | - | - | - | - | - | - | - | C3 | - | Elitek® or R-CHOP |
|  | - | - | - | - | - | - | - | - | C4 | 42 | Elitek® or R-CHOP |
|  | 22.10 | 9.50 | 6.04 | 19.74 | 4.08 | 11.83 | 17.05 | 2 | C5 | 63 | R-CHOP |
|  | 13.48 | 14.74 | 7.47 | 15.43 | 8.13 | 7.35 | 12.47 | 3 | C6 | 84 | R-CHOP |
|  | - | - | - | - | - | - | - | - | End of Chx | 130 | - |
|  | 30.98 | 15.15 | 11.52 | 29.83 | 10.92 | 15.49 | 27.29 | 4 | Radiation | 210 | Radiation |
|  | - | - | - | - | - | - | - | - | - | 224 | End of Radiation |
| **Patient #2** | - | - | - | - | - | - | - | - | T1, C1 | 1 | R-EPOCH, elitek, R-CHOP |
|  | 15.66 | 10.15 | 12.15 | 12.45 | 7.75 | 8.83 | 10.36 | 1 | T1, C2 | 153 | R-EPOCH, elitek, R-CHOP |
|  | - | - | - | - | - | - | - | - | T2, C1 | 216 | Rituximab + Methotrexate + Temodar® |
|  | 18.82 | 9.44 | 6.81 | 18.91 | 3.99 | 10.21 | 22.49 | 2 | T2, C2 | 223 | Rituximab + Methotrexate + Temodar® |
|  | 21.88 | 10.32 | 5.88 | 25.11 | 5.21 | 12.62 | 22.08 | 3 | T3 | 307 | Ruxience |
|  | - | - | - | - | - | - | - | - | T4 | 314 | Temodar® |
|  | 25.62 | 10.64 | 5.77 | 29.29 | 4.21 | 14.25 | 19.11 | 4 | - | 321 | - |
|  | 216.37 | 60.86 | 25.78 | 175.85 | 18.09 | 84.40 | 109.63 | 5 | - | 469 | - |

**Supplementary Table 5**

| **Position and Modification** | **Sequence** | **Sum formula** |
| --- | --- | --- |
| H3 1:8 | H-ARTKQTA-R | C54H91N21O20 |
| H3 1:8 K4Ac | H-ART-Lys(Ac)-QTA-R | C56H93N21O21 |
| H3 1:8 K4Me1 | H-ART-Lys(Me)-QTA-R | C55H93N21O20 |
| H3 1:8 K4Me2 | H-ART-Lys(Me2)-QTA-R | C56H95N21O20 |
| H3 1:8 K4Me3 | H-ART-Lys(Me3)-QTA-R | C57H98N21O20 |
| H3 7:17 | H-ARKSTGGKAP-R | C63H106N24O22 |
| H3 7:17 K9Ac | H-AR-Lys(Ac)-STGGKAP-R | C65H108N24O23 |
| H3 7:17 K9Me1 | H-AR-Lys(Me)-STGGKAP-R | C64H108N24O22 |
| H3 7:17 K9Me2 | H-AR-Lys(Me2)-STGGKAP-R | C65H110N24O22 |
| H3 7:17 K9Me3 | H-AR-Lys(Me3)-STGGKAP-R | C66H113N24O22 |
| H3 7:17 K14Ac | H-ARKSTGG-Lys(Ac)-AP-R | C65H108N24O23 |
| H3 7:17 K9Ac+K14Ac | H-AR-Lys(Ac)-STGG-Lys(Ac)-AP-R | C67H110N24O24 |
| H3 16:26 | H-PRKQLATKAA-R | C70H119N25O22 |
| H3 16:26 K18Ac | H-PR-Lys(Ac)-QLATKAA-R | C72H121N25O23 |
| H3 16:26 K23Ac | H-PRKQLAT-Lys(Ac)-AA-R | C72H121N25O23 |
| H3 16:26 K18Ac+ K23Ac | H-PR-Lys(Ac)-QLAT-Lys(Ac)-AA-R | C74H123N25O24 |
| H3 25:40 | H-ARKSAPATGGVKKPH-R | C88H146N32O27 |
| H3 25:40 K27Ac | H-AR-Lys(Ac)-SAPATGGVKKPH-R | C90H148N32O28 |
| H3 25:40 K27Me1 | H-AR-Lys(Me)-SAPATGGVKKPH-R | C89H148N32O27 |
| H3 25:40 K27Me2 | H-AR-Lys(Me2)-SAPATGGVKKPH-R | C90H150N32O27 |
| H3 25:40 K27Me3 | H-AR-Lys(Me3)-SAPATGGVKKPH-R | C91H153N32O27 |
| H3 25:40 K36Ac | H-ARKSAPATGGV-Lys(Ac)-KPH-R | C90H148N32O28 |
| H3 25:40 K36Me1 | H-ARKSAPATGGV-Lys(Me)-KPH-R | C89H148N32O27 |
| H3 25:40 K36Me2 | H-ARKSAPATGGV-Lys(Me2)-KPH-R | C90H150N32O27 |
| H3 25:40 K36Me3 | H-ARKSAPATGGV-Lys(Me3)-KPH-R | C91H153N32O27 |
| H3 25:40 | H-ARKSAPSTGGVKKPH-R |  |
| H3 25:40 K27Ac | H-AR-Lys(Ac)-SAPSTGGVKKPH-R |  |
| H3 25:40 K27Me1 | H-AR-Lys(Me)-SAPSTGGVKKPH-R |  |
| H3 25:40 K27Me2 | H-AR-Lys(Me2)-SAPSTGGVKKPH-R |  |
| H3 25:40 K27Me3 | H-AR-Lys(Me3)-SAPSTGGVKKPH-R |  |
| H3 25:40 K36Ac | H-ARKSAPSTGGV-Lys(Ac)-KPH-R |  |
| H3 25:40 K36Me1 | H-ARKSAPSTGGV-Lys(Me)-KPH-R |  |
| H3 25:40 K36Me2 | H-ARKSAPSTGGV-Lys(Me2)-KPH-R |  |
| H3 25:40 K36Me3 | H-ARKSAPSTGGV-Lys(Me3)-KPH-R |  |
| H3 39:49 | H-HRYRPGTVAL-R | C75H117N27O22 |
| H3 52:63 | H-RRYQKSTELLI-R | C85H140N28O27 |
| H3 52:63 K56Ac | H-RRYQ-Lys(Ac)-STELLI-R | C87H142N28O28 |
| H3 52:63 K56Me1 | H-RRYQ-Lys(Me)-STELLI-R | C86H142N28O27 |
| H3 52:63 K56Me2 | H-RRYQ-Lys(Me2)-STELLI-R | C87H144N28O27 |
| H3 52:63 K56Me3 | H-RRYQ-Lys(Me3)-STELLI-R | C88H147N28O27 |
| H3 62:69 | H-IRKLPFQ-R | C66H105N21O18 |
| H3 71:83 | H-VREIAQDFKTDL-R | C86H136N26O30 |
| H3 71:84 K79Ac | H-VREIAQDF-Lys(Ac)-TDL-R | C88H138N26O31 |
| H3 71:85 K79Me1 | H-VREIAQDF-Lys(Me)-TDL-R | C87H138N26O30 |
| H3 71:86 K79Me2 | H-VREIAQDF-Lys(Me2)-TDL-R | C88H140N26O30 |
| H3 71:87 K79Me3 | H-VREIAQDF-Lys(Me3)-TDL-R | C89H143N26O30 |
